# Clinical characteristics and surgical management of facial infiltrating lipomatosis: a single center experience

**DOI:** 10.1186/s13005-024-00412-6

**Published:** 2024-02-20

**Authors:** Hongrui Chen, Bin Sun, Wenwen Xia, Yajing Qiu, Wei Gao, Chen Hua, Xiaoxi Lin

**Affiliations:** 1 Department of Plastic & Reconstructive Surgery, Shanghai Ninth People’s Hospital, Shanghai Ninth People’s Hospital, affiliated to Shanghai Jiao Tong University School of Medicine, 639 Zhizaoju Road, Shanghai, 200011 P.R. China; 2 Department of Pathology, Shanghai Ninth People’s Hospital, affiliated to Shanghai Jiao Tong University School of Medicine, Shanghai, China

**Keywords:** Facial infiltrating lipomatosis, Phenotype, Radiological finding, Hemimegalencephaly, Surgery, PIK3CA mutations

## Abstract

**Background:**

Facial infiltrating lipomatosis (FIL) is a rare condition characterized by congenital facial enlargement. Beyond its impact on physical appearance, FIL can also impair essential facial functions such as swallowing, chewing, vision, and breathing, imposing a substantial physiological and psychological burden. Currently, fewer than 80 cases of FIL have been reported, and the characteristics and management strategies for FIL remain unclear.

**Methods:**

We reviewed the clinical, surgical, and radiological records of 39 FIL patients who were treated at our center. Of these, genetic testing was performed for 21 patients.

**Results:**

Aberrant overgrowth involves subcutaneous fat, bones, muscles, glands, tongue, lips, and teeth. Epidermal nevi could be observed in the dermatomes innervated by the three branches of the trigeminal nerve, with the highest frequency seen in the dermatome of the mandibular branch. Four patients exhibited concurrent hemimegalencephaly (HMEG), with one case presenting HMEG on the opposite side of the FIL. Nineteen patients were confirmed to harbor the PIK3CA mutation. Thirty-three patients underwent surgical procedures, with a post resection recurrence rate of approximately 25%.

**Conclusions:**

A variety of maxillofacial structures may be involved in FIL. PIK3CA mutations are important pathogenic factors. Emerging targeted therapies could present an additional treatment avenue in the future. However, surgery currently remains the predominant treatment choice for FIL. The timing and modality of surgery should be individually customized, taking into account each patient's unique circumstances. Notably, there is a significant possibility of postoperative recurrence during childhood and adolescence, necessitating early strategic planning of disease management.

**Supplementary Information:**

The online version contains supplementary material available at 10.1186/s13005-024-00412-6.

## Background

Facial infiltrating lipomatosis (FIL), also known as congenital infiltrating lipomatosis of the face, was first described by Slavin in 1983 as a rare form of facial developmental malformations [1]. FIL manifests as a spectrum of craniofacial conditions, ranging from subtle facial enlargement to severe hyperplasia of adipose tissue, often accompanied by skeletal and dental anomalies [2]. Histological examinations reveal infiltration of non-encapsulated mature adipocytes into the surrounding soft tissues, often associated with increased vascularity and nerve bundles [3]. Although FIL is predominantly asymptomatic, anatomical irregularities can adversely affect the patient's appearance and facial functionality, including impairments to chewing, swallowing, and vision. [4]. These abnormalities can disrupt daily activities and impact psychosocial well-being, necessitating surgical interventions to improve facial morphology [3]. Both debulking and liposuction serve as viable therapeutic options, with the choice largely dependent on the size of the lesion, facial symmetry, patient age, and patient preferences [5]. Despite the benign nature of hyperproliferative adipocytes, their extensive infiltration complicates complete surgical excision, resulting in a high recurrence rate [1].

FIL is primarily sporadic, with no known familial cases, indicating that its pathogenesis is likely driven by somatic variants. The advent of next-generation sequencing (NGS) has markedly advanced our understanding of the underlying mechanisms of overgrowth disorders [6]. In 2014, the identification of somatic mutations in the phosphatidylinositol 3-kinase catalytic subunit alpha (PIK3CA) gene in abnormal tissues from six FIL patients shed light on the molecular etiology of FIL [7]. Subsequent research have consistently confirmed PIK3CA mutations as the causal factor for FIL [8–11]. PIK3CA encodes the p110α catalytic subunit of class IA phosphoinositide 3-kinases (PI3Ks). Studies suggest that PIK3CA mutations can cause hyperactivation of the PI3K-AKT-mTOR signaling pathway, which plays a crucial role in cell proliferation, differentiation, and survival. This hyperactivation, in turn, promotes excessive cell proliferation and tissue overgrowth [12].

Despite numerous reports on FIL's clinical manifestations, debates surrounding its complex characteristics, surgical interventions, and prognosis continue. To address these knowledge gaps, we evaluated the phenotypic features and surgical management of 39 cases and conducted genetic analyses on 21 of these cases. Through this comprehensive approach, we aim to enhance the understanding of this rare disease within the medical community.

## Methods

### Study subjects

"We reviewed patients diagnosed with FIL at Shanghai Ninth People’s Hospital between 2014 and 2022. The diagnosis was confirmed through a comprehensive assessment of each patient's medical history, physical examination, and imaging results. The essential diagnostic criteria included: (1) Unilateral facial enlargement that was either congenital or noted shortly after birth. (2) Magnetic resonance imaging (MR) revealed infiltration of adipose tissue into surrounding muscles and glands. Additional features that supported the diagnosis included: (1) The patient presented with associated macrodontia, hemimacroglossia, epidermal nevi, and thickened lips. (2) PIK3CA mutations were detected in tissue samples obtained through biopsy or surgical excision. Exclusion criteria of this condition were: (1) The patient presented with facial enlargement, but MR primarily revealed a vascular component, such as hemangioma or vascular malformation. (2) There was only unilateral subcutaneous facial adipose hypertrophy, without infiltrative property. Written consent for the use of their clinical data was obtained from all patients. This study was approved by the Institutional Review Board of Shanghai Ninth People’s Hospital. All authors were fully aware of and adhered to the ethical principles outlined in the WMA Declaration of Helsinki for Medical Research Involving Human Subjects, ensuring that the present study strictly complied with the declaration.

### Imaging modalities

MR examinations were performed using a MAGNETOM Verio with a magnetic field strength of 3.0 Tesla. The imaging protocol for maxillofacial MR included axial and coronal T1-weighted images (T1WI), axial and coronal T2-weighted images (T2WI), and axial and coronal T1-weighted images with fat suppression post-contrast. The cranial MR protocol included axial and sagittal T1WI, axial T2WI, and axial FLAIR sequences.

### Treatment

Skeletal reconstruction was conducted using computerized tomography (CT), while MR aided in visualizing soft tissue and fatty infiltrations. Liposuction and/or debulking procedures were employed to remove excess adipose tissue, tailoring the specific approach to each patient's individual characteristics. Preoperative evaluation relied on imaging scans and bone reconstruction models, which effectively depicted both soft and hard facial structures. Given that patients often presented with hemimacroglossia, lip thickening, and osseous hyperproliferation, surgicaladjustments were occasionally conducted to address these specific areas, potentially in a segmented manner.

### Next-generation sequencing

We performed target panel sequencing using high-depth NGS technology in 21 patients. The panel included genes associated with vascular malformations and overgrowth disorders: ACVRL1, GLMN, PIK3CA, AKT1, mTOR, PTEN, MAP2K1, TEK, MAP3K3, GNA11, GNA14, GNAQ, RASA1, EPHB4, SMAD4, STAMBP, KDR, FLT4, KRAS, and BRAF. Genomic DNA was extracted from epidermal nevus (*n* = 3) and subcutaneous adipose tissue (*n* = 18). The sequencing method has been described in a previous article [13]. Briefly, the Qiagen DNA Extraction Kit (#13,323) was utilized to extrate DNA from the tissue samples. Necessary splice adjustments were carried out on the obtained genomic DNA fragments in preparation for sequencing, achieved using the NEBNext Ultra II DNA Library Prep Kit for Illumina. High-throughput sequencing was subsequently conducted on the Illumina Nova Seq 6000 platform upon successful library development. The panel exhibited an average sequencing depth of 10,000 × with coverage exceeding 98%. Analysis of the DNA sequences from the test samples was performed by comparison with the reference sequence hg19 (GRCh37) to identify respective mutations.

## Results

### Phenotypic features

This study included 39 eligible patients (Supplement materials 1), with their demographic data and clinical presentations summarized in Table 1. FIL exhibited a higher prevalence in females, with a male-to-female ratio of 1:1.17. All patients were children or young adults (≤ 25 years old), except for one 43-year-old woman. In 35 individuals, unilateral facial enlargement was observed at birth, while in four patients, facial asymmetry became apparent after one year of age. Notably, all patients in this cohort denied having a family history of FIL or any other form of overgrowth disorder.

**Table 1:** Demographic data and clinical features of patients with FIL

Characteristic	Value
**Gender**	**n (%)**
Male	18 (46.2%)
Female	21 (53.8%)
**Side**	
Left	17 (43.6%)
Right	22 (56.4%)
**Age (years)**	**12.1 ± 9.9**
Range	1–43
**First noticed**	**n (%)**
Birth	35 (89.7%)
≥ 1 year	4 (10.3%)
**Clinical feature**	**n (%)**
Epidermal nevus	16 (41.0%)
Macrodontia	14 (35.9%)
Teeth missing	10 (25.6%)
Hemimacroglossia	20 (51.3%)
Enlarged lingual papilla	6 (12.8%)
Thickened lip	26 (66.7%)

**Fig. 1:**
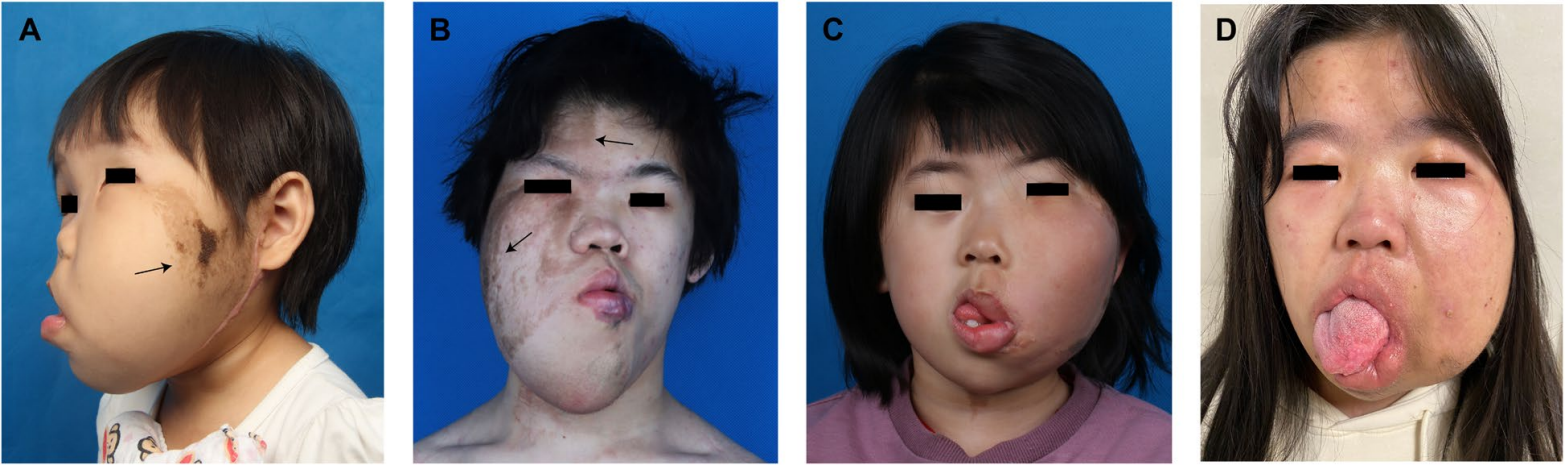
Common clinical presentations of FIL. **A** Patient 28 presented with EN (black arrow) distributed in the V2 and V3 dermatomes of trigeminal nerve. **B** Patient 6 exhibited EN (black arrow) distributed in the V1, V2, and V3 dermatomes of the trigeminal nerve. **C** Patient 30 displayed facial features characterized by thickening of both upper and lower lips, extending beyond the midline but not involving the entire lip. **D** Patient 14 presented with hemimacroglossia and thickening of the lower lip on the affected side

Sixteen patients presented with epidermal nevi (EN). The EN lesions were predominantly brown or black in color, with three patients also exhibited hirsutism within the pigmented area. In all cases, the EN lesions were localized on the affected side, with the exception of Patient 19, who additionally had an EN lesion in the contralateral frontal region. Referring to the relationship between the distribution of port-wine stain and the dermatomes of the trigeminal sensory branch [14], we also analyzed the distribution of EN in the trigeminal dermatomes in the FIL (Table 2). All 16 cases were plotted according to the topographic properties of facial EN. One (6.3%) had isolated ophthalmic branch (V_1_) involvement, seven (43.8%) had isolated mandibular branch (V_3_) involvement, one (6.3%) had combined ophthalmic and maxillary branch (V_1_, V_2_) involvement, six (37.5%) had maxillary and mandibular branch (V_2_, V_3_) involvement (Fig. 1A), and one (6.3%) had all three branches (V_1_, V_2_ and V_3_) included (Fig. 1B). The distribution of EN involving V_3_ was the most frequent (*n* = 14, 87.5%), while the distribution involving V_1_ was relatively rare (*n* = 3, 18.8%).

**Table 2:** Trigeminal dermatome distribution in FIL patients with EN

Patient No	EN distribution on trigeminal dermatome
1	V_3_
3	V_3_
4	V_1_
6	V_1_V_2_V_3_
7	V_3_
10	V_2_V_3_
13	V_2_V_3_
15	V_3_
19	V_1_V_2_
22	V_2_V_3_
27	V_3_
28	V_2_V_3_
30	V_2_V_3_
31	V_3_
32	V_3_
34	V_2_V_3_

Asymmetrical lips were observed in 26 (66.7%) patients. A review of photographs provided by the guardians indicated that although lip thickening was not prominent at birth, it tended to progress with age, becoming evident by the age of one. Lower lip thickening was observed more commonly, with 17 patients presenting with isolated lower lip thickening, two with isolated upper lip thickening, and seven with both lips affected (Fig. 1C). Hyperplasia of the soft tissue, primarily observed on the affected side, occasionally crossed the midline of the lip. There were no instances of complete involvement of either the upper or lower lip. Oral examination revealed macrodontia on the affected side in 14 patients and missing permanent or deciduous teeth in another 10. Hemimacroglossia was identified in 20 patients (Fig. 1D), consistently confined to the affected side, with an enlarged lingual papilla observed in six of these individuals.

### Imaging findings

**Fig. 2:**
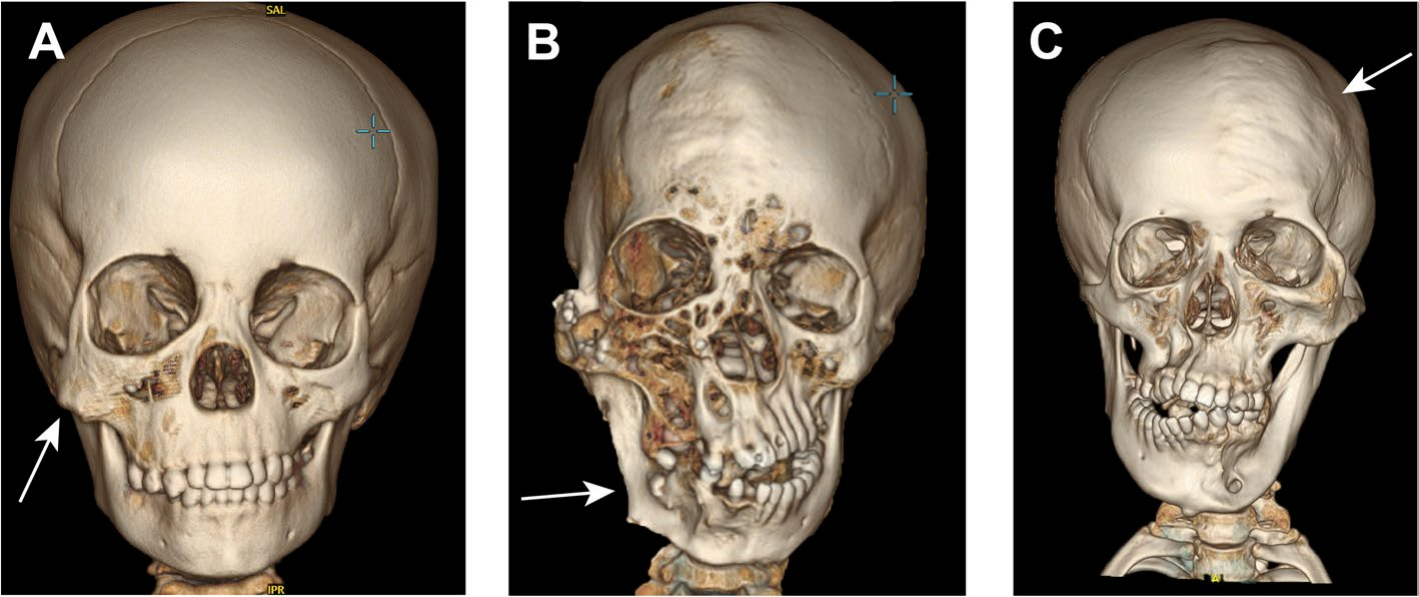
Skeletal deformity in FIL. **A** Bone reconstruction (BR) of Patient 38 revealed hypertrophy of the zygomatic arch and maxilla on the affected side. **B** Bone reconstruction of Patient 1. Disrupted development of the maxilla and mandible due to compression by adipose tissue resulted in dental misalignment. **C** BR of Patient 12 exhibited localized hyperplasia of the frontal bones

Thirty-four individuals underwent CT examinations, and 33 underwent MR examinations. Analysis of skeletal reconstruction revealed the zygoma as the most commonly affected site, with overgrowth noted in 26 patients (66.7%). The excess bone growth primarily occurred in an outward and inferior direction, contributing to the upper facial enlargement (Fig. 2A). The mandible was the second most frequently involved site. In addition to significant overgrowth of the mandibular body, anomalies in the caput mandibulae, collum mandibulae, and coronoid process on the affected side were observed. Maxillary hyperplasia or malformation could induce malalignment of the upper dentition. In cases where both maxillary and mandibular malformations were present, complications such as malocclusion and underbite could arise (Fig. 2B). Frontal bone deformities was observed in two severely affected patients (Patient 1 and Patient 15), with the affected side showing anterior displacement compared to the contralateral side (Fig. 2C). A comparison of skeletal reconstruction images from selected patients during infancy and childhood revealed that, in contrast to the noticeable fat accumulation present at birth, abnormal hyperostosis often remained inconspicuous during infancy, becoming more evident with age.

**Fig. 3:**
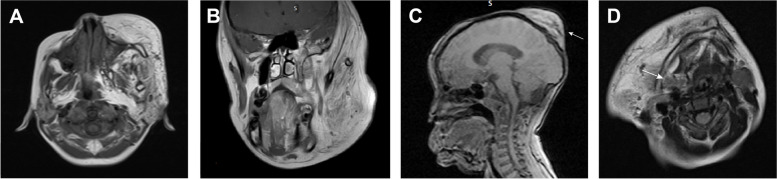
Maxillofacial imaging findings. **A** T1-weighted axial image of patient 30. The patient exhibited evident subcutaneous adipose tissue proliferation, with infiltration observed in the temporalis muscle, masseter muscle, and parotid gland, resulting in the loss of normal boundaries. **B** T1-weighted coronal image of patient 12. Extensive fatty tissue with wide-ranging borders infiltrated superiorly into the temporal muscle. **C** Sagittal image of patient 1. A soft tissue mass was observed on the affected side of the scalp (white arrow), displaying fat signal. **D** T2-weighted axial image of patient 18. The submandibular gland of the patient was also affected by fatty infiltration (white arrow)

MR imaging revealed that the masseter muscle was the most commonly affected masticatory muscle, with increased size and adipose tissue infiltration within the masseter muscle observed in 31 patients (79.5). The medial pterygoid and lateral pterygoid muscles were frequently involved, possibly due to their deeper anatomical locations (Fig. 3A). The temporalis muscle was affected in only three cases (Fig. 3B). Additionally, Patient 1 and Patient 15 also presented with a subcutaneous fat mass on the scalp (Fig. 3C). Owing to its close proximity to the subcutaneous fat, the parotid gland was also highly susceptible to involvement, showing hypertrophy and anterior fat signals. Signs of submandibular gland involvement were observed (Fig. 3D), with a common feature being that the inferior border of the lipomatosis was lower than the base of the mandible (Table 3).

**Table 3:** Affected soft and hard tissue

Anatomical site	Value
**Bone**	
Zygoma	26 (66.7%)
Maxilla	12 (30.8%)
Mandible	15 (38.5%)
Frontal bone	2 (5.1%)
**Soft tissue**	
Masseter	31 (79.5%)
Medial pterygoid	11 (28.2%)
Lateral pterygoid	14 (35.6%)
Temporalis	3 (7.7%)
Parotid gland	34 (87.2%)
Submandibular gland	12 (30.8%)
**Brain**	
Hemimegalencephaly	4 (10.2%)

**Fig. 4:**
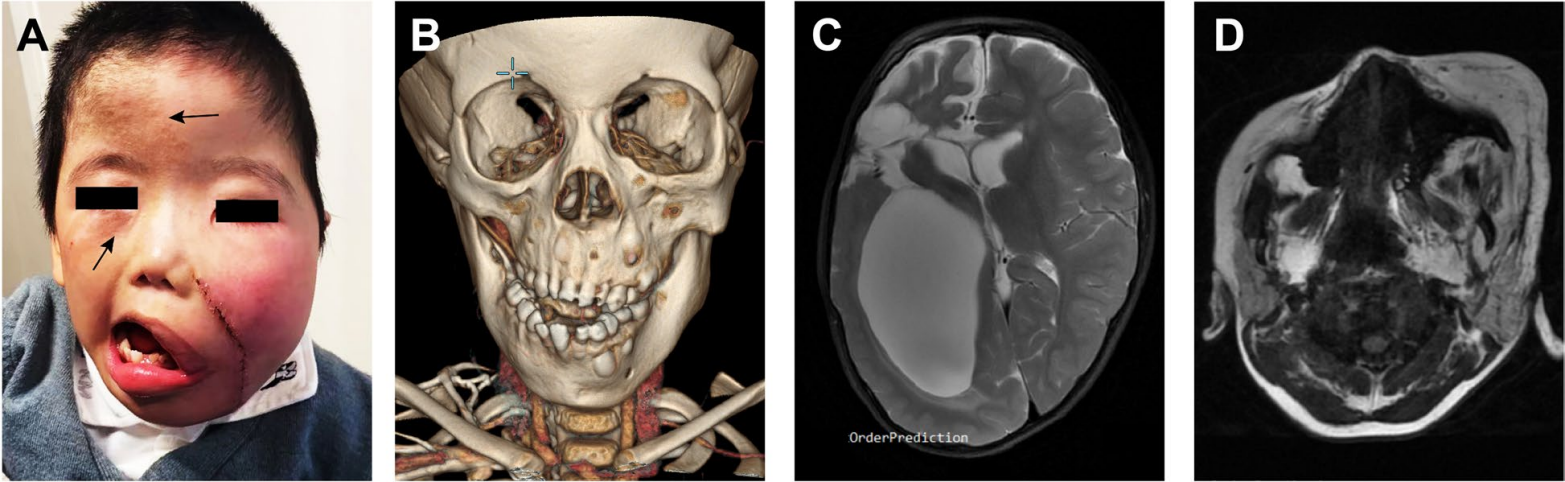
Phenotypic features of Patient 19. **A** Facial appearance was assessed. Epidermal nevi were observed in the right frontal and infraorbital regions. Despite undergoing debulking surgery at another center, facial asymmetry was still conspicuous. **B** BR revealed abnormalities in the zygomatic bone, maxilla, and mandible, along with dental misalignment. **C** T2-weighted axial image demonstrated hemimegalencephaly on the right side. The patient had previously undergone surgery for epilepsy. **D** T1-weighted axial image revealed extensive infiltration of fat into the soft tissues on the left side

Given the frequent association between Hemimegalencephaly (HMEG) and FIL [15], our MR examination also included the brain. We identified four patients with hemispheric enlargement (Patients 2, 6, 19, and 34). In the reported cases of HMEG coexisting with FIL, both conditions were found on the same side. However, in one case (Patient 19), the enlarged hemisphere was contralateral (right) to the FIL (left). Moreover, Patient 19 exhibited epidermal nevi in the right frontal and infraorbital regions (Fig. 4A-D). Patient 6, who had a history of childhood epilepsy, was managed with antiepileptic medication. Due to recurrent seizures, Patient 19 underwent a hemispherectomy. The remaining two patients with HMEG did not exhibit any clinical symptoms.

### Genotypes

Twenty-one patients underwent genetic testing, with the genotypes of 18 patients previously described in our prior investigation [13]. The remaining three patients carried PIK3CA p.Asn345Lys, p.Cys420Arg, and p.Glu453Lys mutations. Overall, the detection rate of PIK3CA mutations in our patients was 19/21 (90.5%), highlighting a correlation between PIK3CA mutations and the development of FIL.

### Surgical management

**Fig. 5:**
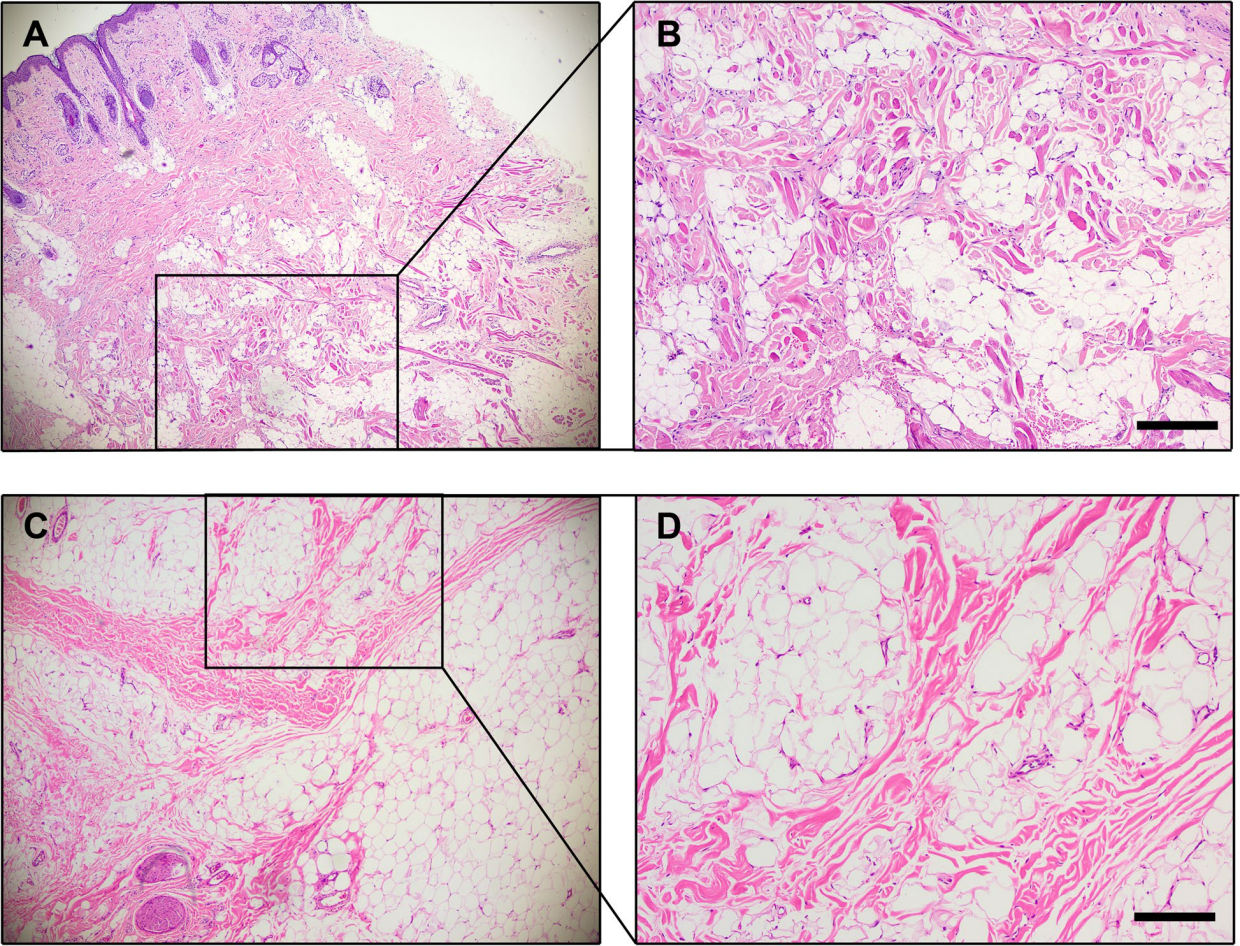
Pathologic findings in FIL. **A**, **B** Histological findings of patient 18. **C**, **D** Histological findings of patient 17. Microscopic images revealed infiltration of mature adipocytes into subcutaneous tissues and skeletal muscle fibers (Hematoxylin and eosin staining). Scale bar: 100 μm

Thirty-three patients underwent surgical treatment at our center. Among these, thirteen patients required multiple surgical procedures: eight underwent debulking or liposuction due to postoperative recurrence, and five needed subsequent adjustments to address complications such as scarring and lip hypertrophy. The overall postoperative recurrence rate was 24.2%. Histopathological examination revealed infiltration of mature adipocytes into adjacent tissues, including subcutaneous mucosa, epidermis, elastic fibers, and glands (Fig. 5). Notably, of the eight patients who experienced recurrence, seven had undergone initial surgery during childhood. In contrast, no recurrence was observed in patients who underwent surgery during adulthood. In FIL, major concerns involve abnormal proliferation of fat, bones, tongue, and lips, leading to characteristic phenotypic features like facial asymmetry, lip thickening, and hemimacroglossia. These abnormalities mandate careful evaluation prior to any surgical intervention, and detailed consultations should be held with parents regarding potential postoperative outcomes, such as recurrence, increased risk of scarring in patients harboring PIK3CA mutations [16], and facial nerve damage. Each case's unique findings should be individually evaluated.

**Fig. 6:**
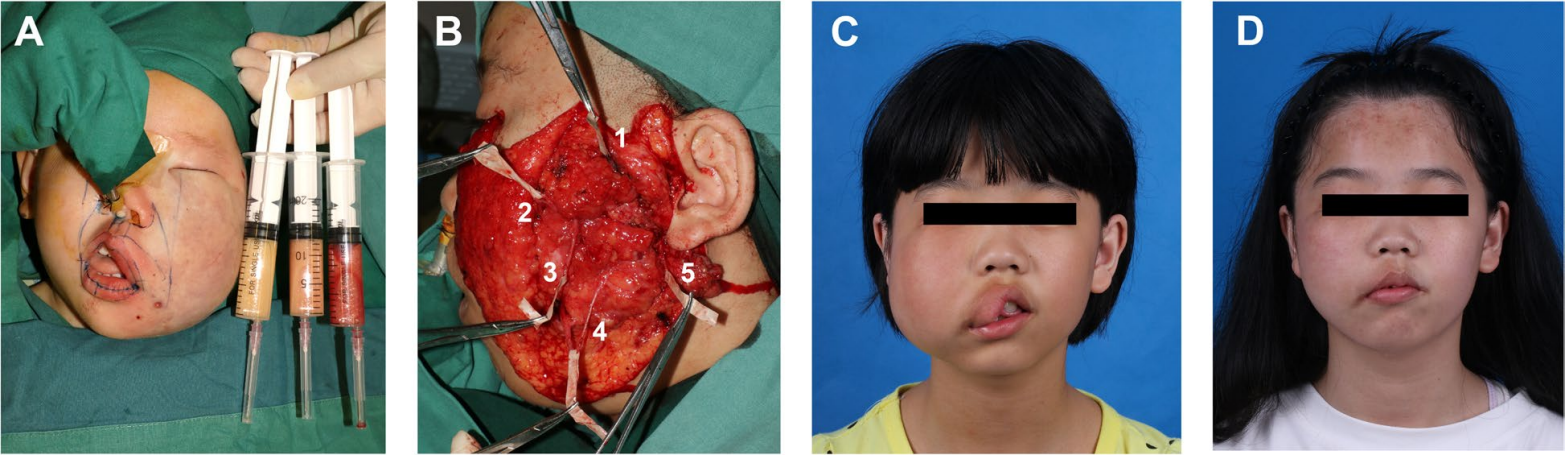
Surgery-related photos. **A** Patient 27 underwent liposuction. **B** Patient 7 underwent facial nerve dissection to protect the facial nerve while removing excess adipose tissue. The branches of the facial nerve were: 1: frontal branch, 2: zygomatic branch, 3: upper buccal branch, 4: lower buccal branch, 5: cervical branch. **C** Preoperative photo of the Patient 22 at 11 years old. **D** The Patient 22 underwent debulking and upper lip adjustment at the age of 13. This was her postoperative photo at the age of 15. There has been significant improvement in her facial contour and lip morphology

For children and adolescents (under 14 years old) whose facial structures had not yet fully matured, liposuction was commonly employed [5]. The total aspiration volume varied case by case, depending on the patient's age, body size and estimated final aspiration volume (Fig. 6A). However, for pediatric patients with severe phenotypic manifestations, liposuction was not applicable, and we instead resorted to invasive debulking combined with facial nerve dissection to remove excessive adipose tissue (Fig. 6B). For adult patients, we recommended a more comprehensive approach to achieve symmetrical facial contours. Osteotomy was employed for patients with maxillary and mandibular hypertrophy, with the amount of bone to be removed personalized for each case. Effective dissection of the facial nerve followed by reductive plasty and successful reduction of the mandibular angle is particularly important for contour change, but facial nerve dissection may only be performed once and is not appropriate in infancy or childhood. Removal of excess tissue was performed as needed to reshape the tongue or lips (Fig. 6C, D).

## Discussion

This study describes an analysis of a clinical cohort of 39 patients with FIL. To our knowledge, this is the largest number of cases reported by a single center to date. In these cases, left-sided disease was less common than right. Most patients exhibited facial asymmetry at birth, while a minority showed more noticeable enlargement after one year of age. Female patients outnumbered males, and all cases were nonhereditary. PIK3CA, a widely expressed lipid kinase, regulates signaling pathways associated with cell proliferation, movement, survival, and metabolism [17]. Somatic PIK3CA missense mutations were detected in the subcutaneous tissue of FIL patients [7], and subsequent studies identified PIK3CA hotspot mutations in various types of FIL tissues (skin, mucosa, adipose, bone) [8], confirming postzygotic PIK3CA mutations as the underlying cause of FIL. Genetic testing was performed on lesion tissues of 21 patients in our center, contingent upon patient preferences and the timing of the introduction of NGS. Nineteen specimens were found to harbor somatic PIK3CA mutations. Furthermore, our previous analysis [13], revealed that hotspot mutations were associated with more severe FIL phenotypes, further enriching our understanding of FIL's molecular pathogenesis.

EN is common in our center's patients. We found that approximately 40% of FIL patients had EN on the affected side, distributed across the dermatomes innervated by the three branches of the trigeminal nerve. The highest frequency was observed in the mandibular branch (V3) dermatome. Three patients underwent genetic sequencing using EN specimen, with two patients (patients 4 and 6) found to harbor the PIK3CA p.Cys420Arg mutation. We recommend using EN specimens or subcutaneous adipose tissue for genetic testing in patients who are not prepared for open surgery. The reasons are as follows: (1) both epidermal nevi and subcutaneous adipose tissue are directly involved in the disease process. Changes in these tissues are often visible or palpable, making them readily accessible for sampling, (2) these tissues are expected to carry disease-associated genetic alterations. Sampling from these tissues, therefore, provides us with a good chance of detecting disease-related genetic mutations. (3) DNA extraction from these tissues is relatively straightforward and yields high-quality DNA suitable for NGS.

CT bone reconstruction revealed varying degrees of hyperplasia and deformity in the affected maxillofacial bones, with pronounced abnormalities in the zygomatic bones. Our observations mirrored Padwa's findings [2], where hyperplasia of adipose tissue could be discerned at an early stage, and potential skeletal deformities gradually became apparent over time. Notably, in two patients, the affected side of the maxillofacial bones exhibited a pronounced anterior displacement compared to the contralateral side. This resulted in facial asymmetry, not only in the coronal plane but also in an anterior–posterior orientation within the sagittal plane.

MR can accurately identify and diagnose the adipose nature of FIL, thus excluding potential confounding vascular malformations and obviating the need for histological examination [18]. In our cohort, MR imaging revealed that the masticatory and parotid glands, situated closest to the subcutaneous fat layer, were the soft tissues most prone to involvement, followed by the medial pterygoid, lateral pterygoid, and submandibular glands. The utility of MR in identifying central nervous system lesions is also well established. Even in the absence of neurological features, MR should be employed for further screening for possible HMEG presence when FIL is suspected or diagnosed. The occurrence of HMEG is associated with various cortical developmental disorders, encompassing neuronal differentiation, proliferation, and apoptosis [19]. To date, no simultaneous genetic testing of adipose and brain tissues has been performed in patients with FIL and concomitant HMEG. However, considering the association of both conditions with PIK3CA mutation [20], it is plausible to hypothesize a shared etiological origin. Notably, all previously reported cases of FIL with concurrent HMEG had lesions on the same side. In contrast, we observed a patient with HMEG and FIL located on opposite sides. While this patient has not yet undergone genetic testing, subsequent molecular examination of the bilateral facial lesions may provide insights into the underlying causes of this unique phenotype.

The co-occurrence of diverse tissue abnormalities, ranging from EN distribution to overgrowth of maxillofacial soft and hard tissues, coupled with potential hemimegalencephaly, has stimulated our exploration into the underlying etiology of FIL. Prasad proposed that FIL may result from neurogenic dysregulation and compared the similarities between lipomatosis of nerve (LN) and FIL [21]. LN commonly affects the median nerve in the wrist and palm, resulting in excessive growth within the nerve territory, such as macrodactyly and hemihypertrophy, and is also associated with PIK3CA mutations [22]. In FIL, commonly affected sites such as the masseter muscle, parotid gland, and the most frequent distribution area of EN, are innervated by the maxillary or mandibular branches of the trigeminal nerve. However, histological examination of the nerves in FIL did not reveal fusiform expansion of the nerve with fibroadipose tissue infiltration between nerve bundles seen in LN [22], suggesting that the role of neurogenic abnormalities in FIL remains to be explored. Maclellan hypothesized that FIL may potentially originate from a mutation occurring in the first pharyngeal arch, given its subsequent derivation into the maxilla, mandible, masticatory muscles, and anterior two-thirds of the tongue [7]. Another theory is that FIL may originate from aberrant development and migration of neural crest cells. The affected tissues in FIL predominantly arise from the mesoderm (bone, muscle, fat) and neuroectoderm (brain, epidermal nevi) [12]. The neural crest is responsible for much craniofacial development. After neural tube formation, neural crest cells migrate along established pathways to give rise to structures of mesodermal origin (such as blood vessels, melanocytes, adipose tissue, membranous bone, and connective tissue) throughout the embryo. Additionally, the neural crest appears in a segmental manner in all three primary brain vesicles: rhombencephalon, mesencephalon, and prosencephalon. The prosencephalic neural crest migrates rostrally to the head as a series of vertically oriented cell columns [23]. It has been suggested that adipose tumors are terminal overgrowth arising from dysregulation of multipotent neural crest cells [24]. We speculate that if a small proportion of neural crest cells randomly carry PIK3CA mutations at an early stage, it may lead to overproliferation of maxillofacial tissue from which they differentiate, generating the clinical phenotype of FIL. The fourth potential reason is that cells carrying PIK3CA mutations may exert indirect growth-promoting effects on adjacent or distant cells, which may involve direct cell–cell contact, paracrine signaling molecules, exosomes, changes in extracellular matrix components, and other unknown cell–cell interactions [12]. Further experimental studies are needed to substantiate this hypothesis.

Differential diagnosis of FIL has been extensively discussed in previous literature [3, 25]. Surgery remains the mainstay of treatment for FIL, but there is currently no standardized guideline regarding the timing and approach of surgical intervention. Due to the nonmalignant nature of FIL, complete excision is generally not recommended by most scholars. The optimal timing for operation is still a subject of debate. Slavin advocated for early excision to control excessive growth [1], while Wingerden recommended delayed excision to achieve facial nerve preservation and better symmetry [5]. Padwa advocated for delayed surgery and temporary measures in young patients [2]. We believe that the timing of surgery should be personalized based on the patient's phenotype and severity. For patients with mild facial enlargement, a conservative approach such as liposuction and adjustments to the lips and tongue can be considered. However, for rapidly progressing cases, delaying surgery is impractical as additional facial overgrowth can lead to loss of normal contour of the cheeks, nose, and lips, and may impair functions such as chewing, swallowing, and speech, and even compromise the airway [26]. Furthermore, parents of pediatric patients often request early surgery for aesthetic reasons to prevent their children from experiencing self-esteem issues related to their appearance during their school years. The postoperative recurrence rate in our center was 24.2%, which is lower than the reported data in previous literature, where the highest reported rate reaching 79% [27]. Among all the recurrent cases, 87.5% were children under the age of 14. Padwa suggested that growth hormone may play a role in recurrence, as surgeries performed before the end of adolescence were more likely to experience recurrence [2]. We also agree with this viewpoint, as there have been few complaints of recurrence among adult patients who underwent surgery in our center. Early removal of the lesion may help normalize maxillofacial structures. A previous report found that open bite deformity due to large maxillofacial venous malformations with macroglossia spontaneously improved significantly after sclerotherapy and laser therapy [28]. We found one patient's underbite was naturally corrected after two liposuction procedures, suggesting that relieving the pressure of adipose tissue on the bones may potentially correct occlusal disorders. However, further evidence is required to support this finding.

Assessment of treatment outcomes primarily hinges on clinical manifestations and patient's subjective feedback. This evaluation includes symptom relief and improvements in functional aspects. Additionally, improvements in appearance are assessed by comparing preoperative and postoperative photographs and imaging data to evaluate changes in facial symmetry and contour. With regard to potential complications, monitoring is also in place for any postoperative issues such as infection, pain, and facial nerve damage. Regular follow-ups are conducted to assess whether there is recurrence of the lesion. Currently, there isn't a comprehensive standardized criterion for evaluating the effectiveness of interventions in FIL. Future research may require a larger patient cohort and a more comprehensive evaluation method, such as pain scoring, functional assessment scales, quality of life questionnaires, etc. to accurately assess treatment effects. With the advancement of genetic testing technology, the detection and analysis of genetic mutations may also aid in assessing disease prognosis and treatment response.

Recently, targeted therapies focusing on PIK3CA mutations and downstream signaling pathways have shown promising progress. The PI3K inhibitor alpelisib has been found to reduce the adipose volume of FIL and improve functionality [4]. Additionally, the AKT inhibitor miransertib has been reported to improve the quality of life and seizures in patients with FIL and HMEG [29]. Although the disease cannot be cured, targeted inhibitors may prevent progression or recurrence, but more clinical data are needed to support this perspective.

Limitations of this study include the fact that all data were derived from a single center, which may introduce biases in phenotype assessment and treatment strategies. Additionally, genetic testing was only performed on a subset of newly enrolled patients. Linking genetic mutation analysis with disease classification, diagnosis, and prognosis may provide insights for future treatment of FIL and other overgrowth disorders. Another disease associated with PIK3CA mutations, CLOVES syndrome, has been reported to have an increased risk of developing Wilms tumor [30]. We did not perform systemic examinations, such as hematological and visceral evaluations on our patients. To establish the correlation between mutations and clinical phenotypes and prognosis, a more comprehensive analysis is required, involving a larger number of cases and more advanced methodologies.

## Conclusion

In addition to hyperproliferative fat, maxillofacial muscles, glands, and bone may be involved in FIL. PIK3CA mutations are a key pathogenic mechanism driving the development of FIL. Surgery remains an important approach to correcting maxillofacial deformities, with the specific timing and procedure depending on the unique presentation of the individual case.

### Supplementary Information

**Additional file 1.**

## Data Availability

No datasets were generated or analysed during the current study.

## Abbreviations

FIL: Facial infiltrating lipomatosis
PI3K: Phosphatidylinositol3-kinase
AKT: Protein kinase B
mTOR: Mammalian target of rapamycin
PIK3CA: Phosphatidylinositol 3-kinase catalytic subunit alpha
NGS: Next-generation sequencing
EN: Epidermal nevi
CT: Computed tomography
MR: Magnetic resonance imaging
BR: Bone reconstruction
HMEG: Hemimegalencephaly
CLOVES: Congenital lipoma overgrowth, vascular malformation, epidermal nevi, scoliosis
